# P19 - Assessment of quality of life in Uruguayan children with atopic eczema and their families

**DOI:** 10.1186/2045-7022-4-S1-P74

**Published:** 2014-02-28

**Authors:** Iris S Ale, Ingrid Rivas, Mariela Alvarez

**Affiliations:** 1 Department of Dermatology and Department of Allergology, Republic University of Uruguay, Montevideo, Uruguay

## Background

Skin diseases produce a negative impact on the psycho-emotional state, social relationships and daily activities. This effect should be particularly important during childhood, a critical period for physical and psychosocial development. However, there is a scarcity of publications on the impact of dermatological diseases in the pediatric population.

Atopic eczema (AE) is a chronic eczematous and pruritic skin disease that may affect the physical, social, and emotional functioning of the affected children as well as their families.

## Methods

One hundred and twenty children (69 females and 51 males) from 6 months to 14 years old, attending the Department of Dermatology at the Pediatric University Hospital in Montevideo, Uruguay, with a confirmed diagnosis of AE -according to the criteria of Hanifin and Rajka- and their parents, entered the study. As a control group we included 100 healthy children of comparable age, gender and socioeconomic background, as well as their parents. The data collection instruments were the Children's Dermatology Life Quality Index (CDLQI), the Infant's Dermatitis Quality of Life Index (IDQOL) and the Dermatitis Family Impact Questionnaire (DFI). Severity of AE was assessed with the SCORAD.

## Results

The median score for the IDQOL was 8.92 (range 2-23, n=66), for the CDLQI, 10.84 (0-26, n=54), and for the DFI, 8.17 (4-25. n=120). All these values were significantly different from the control subjects. There was no significant gender difference.

## Conclusion

The results show that AE significantly impairs the children's quality of life in all age groups and also the quality of life of their families. We suggest the regular evaluation of the quality of life in the clinical management of children with AE.

